# Nucleosome Positioning on Episomal Human Papillomavirus DNA in Cultured Cells

**DOI:** 10.3390/pathogens10060772

**Published:** 2021-06-19

**Authors:** Isao Murakami, Takashi Iwata, Tohru Morisada, Kyoko Tanaka, Daisuke Aoki

**Affiliations:** 1 Department of Obstetrics and Gynecology, Keio University School of Medicine, Tokyo 160-8582, Japan; isao0309@gmail.com (I.M.); morisada@a7.keio.jp (T.M.); aoki@z7.keio.jp (D.A.); 2 Department of Obstetrics and Gynecology, Toho University Ohashi Medical Center, Tokyo 153-8515, Japan; kyoko.tanaka@med.toho-u.ac.jp

**Keywords:** human papillomavirus, nucleosome positioning, life cycle, long control region, E6

## Abstract

Several human papillomaviruses (HPV) are associated with the development of cervical carcinoma. HPV DNA synthesis is increased during the differentiation of infected host keratinocytes as they migrate from the basal layer of the epithelium to the spinous layer, but the molecular mechanism is unclear. Nucleosome positioning affects various cellular processes such as DNA replication and repair by permitting the access of transcription factors to promoters to initiate transcription. In this study, nucleosome positioning on virus chromatin was investigated in normal immortalized keratinocytes (NIKS) stably transfected with HPV16 or HPV18 genomes to determine if there is an association with the viral life cycle. Micrococcal nuclease-treated DNA analyzed by Southern blotting using probes against HPV16 and HPV18 and quantified by nucleosome scanning analysis using real-time PCR revealed mononucleosomal-sized fragments of 140–200 base pairs that varied in their location within the viral genome according to whether the cells were undergoing proliferation or differentiation. Notably, changes in the regions around nucleotide 110 in proliferating and differentiating host cells were common to HPV16 and HPV18. Our findings suggest that changes in nucleosome positions on viral DNA during host cell differentiation is an important regulatory event in the viral life cycle.

## 1. Introduction

Papillomaviruses (PVs) are small double-stranded DNA viruses that infect stratified epithelia, causing benign or malignant hyperproliferative lesions with the completion of the human papillomavirus (HPV) life cycle, which is closely linked to the differentiation of keratinocytes. One of the most typical characteristics of PVs is their genotype-specific host restriction and the preference shown by different PV types for distinct anatomical sites [1,2,3]. To date, more than 200 HPV types have been sequenced and are further divided into low- and high-risk types based on the cancer risk associated with their infection [4,5]. Types 16, 18, 31, 33, 34, 35, 39, 45, 51, 52, 58, and 70 are regarded as high-risk HPV types because they are found in cervical carcinomas. HPVs are thought to gain access to the basal cells of the epithelium through small traumas. The virus life cycle is linked to the differentiation of the infected cell as it migrates from the basal layer to the spinous layer of the epithelium. Both the replication of viral DNA and transcription from HPV promoters increase in response to differentiation of the host keratinocytes. However, the mechanism responsible for the increase in HPV DNA synthesis has not been clearly demonstrated.

The nucleosome is the basic unit of eukaryotic chromatin, consisting of a histone core around which DNA is wrapped. Each histone core is composed of two of each of the histone proteins H2A, H2B, H3, and H4. A stretch of approximately 147 bp DNA wraps in 1.67 left-handed turns around the histone octamer to form a nucleosome. The combination of nucleosome positions and their chemical and compositional modifications is critical to genome regulation. The spatial accessibility of the nucleosome-protected sequence differs dramatically from that of the nucleosome-free sequence. Nucleosome occupancy and accessibility are important aspects of chromatin organization, with direct implications for gene regulation and all other DNA-related processes in the cell nucleus. This stereotypical organization has been explained by the presence of barrier complexes that bind to promoters and create an energy barrier, preventing nucleosome formation, but other determinants of nucleosome positions are also known. Therefore, nucleosome positioning and distribution may affect DNA-related cellular processes such as DNA replication, repair, transcription, and recombination, and may be related to the regulation of gene expression and the local mutation rate [6,7,8].

The most frequently used method of mapping nucleosome positions and occupancy involves digestion of chromatin with micrococcal nuclease (MNase), an endo- and exonuclease that preferentially digests the naked DNA between nucleosomes, releases the nucleosomes from chromatin, and enriches the nucleosome-protected DNA fragments. To determine nucleosome positions and occupancy, the resulting undigested DNA is subjected to high throughput sequencing and mapped to the reference genome.

Transcription regulatory mechanisms have been found to play a role in the life cycle of viruses. This is particularly well documented for the DNA genome of the retrovirus mouse mammary tumor virus (MMTV). The long terminal repeat (LTR) of MMTV is organized in the form of six nucleosomes. Mutual influences between one of these nucleosomes and certain transcription factors determine promoter access and transcriptional activation [9,10]. In the SV40, most of the regulatory region is normally free of nucleosomes [11,12]. Nucleosomes can be assembled in vitro that overlap with binding sites for the Sp1 transcription factor in the early promoter, with flanking nucleosomes possibly positioned as a result of neighboring effects [13]. Antagonistic interactions between nucleosomes and Sp1 may reduce the promoter access of the transcriptional apparatus [14,15]. Access of the replication machinery to the SV40 origin of replication is also blocked by nucleosomes, and this can be overcome by nucleosome remodeling in the presence of T antigen [16,17]. In papillomaviruses, the chromatin organization has been established for bovine papillomavirus type 1 (BPV-1) in situ [18,19], but whether nucleosomes persist on BPV-1 DNA packaged in viral particles remains unclear [20]. HPV genomes have been shown to be bound by nucleosomes in an ordered arrangement around the viral promoters [21].

In this study, we used normal immortal keratinocyte (NIKS) cell lines containing HPV16 and HPV18 genomes as cell models to compare the life cycle strategies of HPV. NIKS cells grown in a monolayer switch from a proliferative and undifferentiated proliferation mode at pre-confluence to a differentiated and post-mitotic proliferation mode at post-confluence. HPV comprise 8 kb of double-stranded, circular DNA with eight protein-coding genes (L1 and L2, which encode capsid proteins, and E1, E2, E4, E5, E6, and E7, which encode proteins involved in replication, transcription, and transformation) and the non-coding, regulatory long control region (LCR). E6 is a major oncoprotein, which is involved in tumorigenesis and is highly expressed in tumors. LCR is approximately 850 bp, adjacent to E6 downstream, and contains the early promoter and regulatory elements involved in viral DNA replication and transcription [22]. Therefore, our research focuses on the LCR and part of E6. We report here the nucleosome positioning on HPV16 and HPV18 DNA during the maintenance and propagation of keratinocytes. These results provide insights into the control of the HPV life cycle from the LCR to the E6 gene. This is the first study that has used normal keratinocytes harboring episomal HPV DNA to investigate nucleosome positioning.

## 2. Results

### 2.1. Nucleosome Organization of the HPV Genome and Host Genome

**Figure 1:**
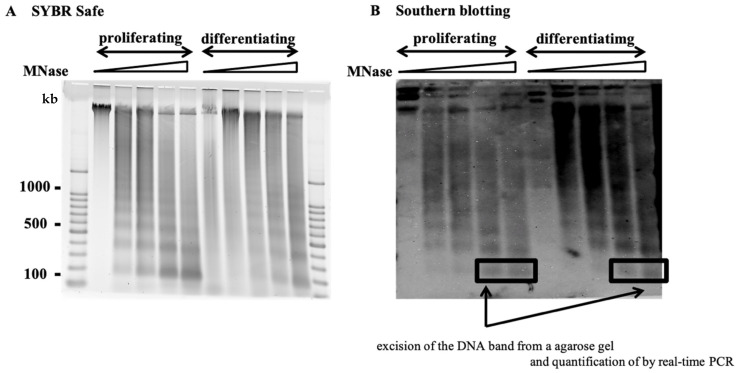
Southern blotting of MNase-treated HPV chromatin. Chromatin from NIKS cells harboring HPV16 or HPV18 was digested with 1–5 U MNase. (**A**) DNA was electrophoretically separated on a 1.5% agarose gel and run at 50 V for 3 h. (**B**) The gel was probed for the full genome of HPV16 or HPV18. Mononucleosome-sized fragments (boxes) were excised from the gel and purified.

HPV16 or HPV18 genome chromatin was digested with 1–5 U MNase. The DNA was electrophoretically separated on a 1.5% agarose gel and stained using SYBR Safe. The MNase-treated chromatin was examined by Southern blotting using probes for full-length HPV16 or HPV18 in transfected NIKS cells. Digestion of the HPV16 and HPV18 genomes using MNase resulted in a non-random cleavage pattern of approximately 200 bp fragments. This finding suggested the presence of specifically localized or phased nucleosomes on HPV16 and HPV18 genome chromatin. Mononucleosome bands (approximately 200 bp) were most evident in the proliferating NIKS cells digested with 1 U and 5 U MNase and differentiating NIKS cells digested with 1 U and 5 U MNase. These bands were excised from the agarose gel and quantified using real-time PCR (Figure 1).

### 2.2. Nucleosome Positioning on Episomal HPV DNA in Cultured Cells

We next performed a nucleosome-scanning experiment using real-time PCR and interpreted the results as follows. First, if a nucleosome was perfectly positioned, a primer pair located completely within this nucleosome would yield a maximal histone density that was defined as 100%. However, if one primer was located within a nucleosome and the other primer was located outside the nucleosome, the apparent nucleosome density would be 0%. Thus, a positioned nucleosomal array would generate peaks and valleys, with the valleys corresponding to linker regions between the nucleosomes. Second, a region occupied by randomly positioned nucleosomes would display a relatively constant level of nucleosome density that was less than the maximal level. Third, a region deficient in nucleosomes would display a low density over the entire region.

**Figure 2:**
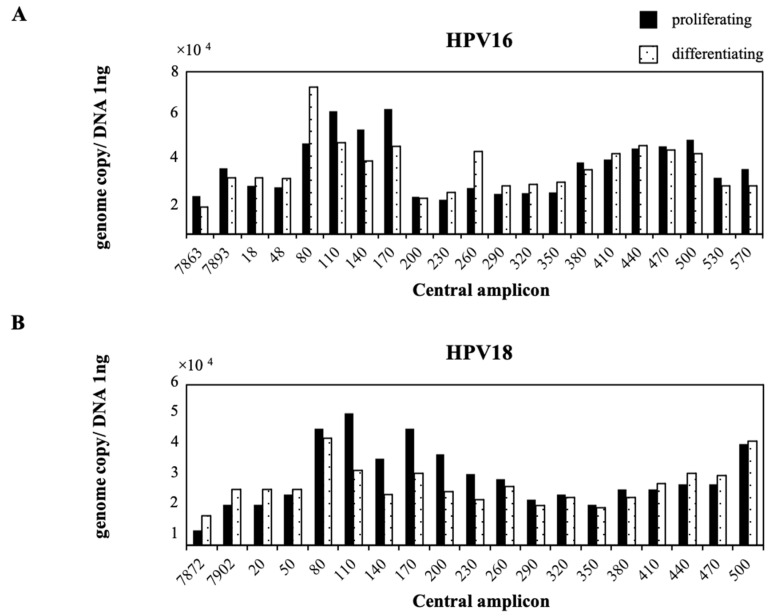
Nucleosome positioning on episomal HPV16/18 DNA in cultured cells. Chromatin and purified genomic DNA were digested with MNase, and mononucleosomal-sized DNA fragments (140–200 bp) were isolated by gel electrophoresis. The mononucleosomal-sized DNA fragments were quantified by real-time PCR. Genome copy number was normalized by 1 ng DNA. The *x*-axis represents the central amplicon of each primer. (**A**) In proliferating NIKS cells harboring HPV16, nt.80–170 and nt.380–500 of HPV16 DNA were relatively MNase-resistant. In differentiating NIKS cells harboring HPV16, nt.80–110 and nt.380–500 of HPV16 DNA became more MNase-resistant and nt.200–350 became less MNase-resistant. (**B**) In proliferating NIKS cells harboring HPV18, nt.80–230 and nt.440–500 of HPV18 DNA were relatively MNase-resistant. In differentiating NIKS cells harboring HPV18, nt.80–110 and nt.440–500 of HPV18 DNA became more MNase-resistant, and nt.200–350 of HPV18 DNA became less MNase-resistant.

We used NIKS cells harboring HPV16 or HPV18 that were cultured under proliferating or differentiating conditions. In proliferating NIKS cells harboring HPV16, nt.80–170 and nt.380–500 of HPV16 DNA were relatively MNase-resistant. In differentiating NIKS cells harboring HPV16, nt.80–110 and nt.380–500 of HPV16 DNA became more MNase-resistant and nt.200–350 became less MNase-resistant (Figure 2A). In proliferating NIKS cells harboring HPV18, nt.80–230 and nt.440–500 of HPV18 DNA were relatively MNase-resistant. In differentiating NIKS cells harboring HPV18, nt.80–110 and nt.440–500 of HPV18 DNA became more MNase-resistant, and nt.200–350 of HPV18 DNA became less MNase-resistant (Figure 2B). Thus, the MNase-resistant regions of HPV DNA changed according to the proliferating or differentiating status of the host cell. Furthermore, a change in the MNase-resistant region around nt.110 between the proliferating and differentiating conditions of the host cell was common in HPV16 and HPV18 DNA.

## 3. Discussion

In this study, we analyzed nucleosome positioning on HPV DNA in NIKS cells harboring HPV16 or HPV18 cultured under proliferating or differentiating conditions. NIKS cells are spontaneously immortalized human keratinocyte cells arising from the BC-1-Ep strain of normal human neonatal foreskin keratinocytes [23]. NIKS cells, which are non-tumorigenic cells, maintain cell type-specific proliferation and differentiation characteristics in monolayer culture. We previously reported a cell model using NIKS cells harboring HPV11 and HPV16 to draw direct comparisons between the life cycle strategies of HPV [24]. In previous studies, we found that the pre-confluent state of NIKS cells exhibited the molecular properties of cell proliferation, while the post-confluent state had the molecular properties of cell differentiation. Therefore, NIKS cells represent an important new tool for the study of proliferation and differentiation in stratified squamous epithelia.

Standard MNase positioning can describe positioned nucleosomes, but it does not easily distinguish whether a given DNA region is occupied by no nucleosomes or randomly positioned nucleosomes. In addition, even when the positioned nucleosomes have been identified, it is difficult to specify the percentage of chromosomes that have a nucleosome at this position. Therefore, we modernized a nucleosome scanning method for positioning nucleosomes and identifying nucleosome density in a quantitative manner. Chromatin and purified genomic DNA were digested with MNase, and mononucleosomal-sized DNA fragments (140–200 bp) were isolated using gel electrophoresis. In the proliferating NIKS cells harboring HPV16, nt.80–170 and nt.380–500 of HPV16 DNA were relatively MNase-resistant. However, in the differentiating NIKS cells harboring HPV16, nt.80–110 and nt.380–500 of HPV16 DNA became more MNase-resistant, and nt.200–350 became less MNase-resistant (Figure 2A). In proliferating NIKS cells harboring HPV18, nt.80–230 and nt.440–500 of HPV18 DNA were relatively MNase-resistant. In differentiating NIKS cells harboring HPV18, nt.80–110 and nt.440–500 of HPV18 DNA became more MNase-resistant, and nt.200–350 of HPV18 DNA became less MNase-resistant (Figure 2B). These results indicated that changes in the MNase-resistant regions around nt.110 between the proliferating and differentiating conditions of the host cell were common to HPV16 and HPV18. This change might represent a general modification that occurs among other HPV types and may serve as an important regulatory function during the viral life cycle that depends on the status of host cell differentiation. In differentiating cells, nt.200–350 of HPV16 and HPV18 DNA became less MNase-resistant, and this change may improve the accessibility of multiple proteins that interact directly with promoter DNA. In this study, we used episomal HPV16 and HPV18 [24]. This might be the reason that differences in MNase-resistant or less MNase-resistant areas were observed between HPV16 and HPV18. Most nucleosomes that exist in eukaryotic and viral chromatin are probably not specifically positioned [25], and most often assume random positions by sliding freely along any particular DNA segment [26,27]. However, the sequences of particular nucleotide segments can affect physical properties such as their curvature, and these properties may determine the precise position of nucleosomes [28,29]. However, nucleosomes were found to occupy specific positions relative to regulatory DNA sequences in the majority of several carefully studied genes. For some of these genes, such as the LTR of MMTV [9,30,31] and *Xenopus* 5S rRNA genes [32], only one or a few nucleosomes are specifically positioned because of the underlying structural properties of the DNA, while neighboring nucleosomes may be arranged in a regular array relative to a specifically positioned nucleosome, thereby also binding a constant segment.

In conclusion, our results suggest that a shift in the position of the nucleosomes in the chromatin organization of HPV16 and HPV18 LCR and E6 is an important regulatory event during the viral life cycle that depends on the status of host cell differentiation.

## 4. Materials and Methods

### 4.1. Cell Culture

In this study, 293T cells (ATCC) were maintained in Dulbecco’s modified Eagle’s medium (DMEM; Sigma, St. Louis, MO, USA) supplemented with 10% fetal bovine serum (FBS; HyClone, Marlborough, MA, USA) and 1% penicillin and streptomycin. NIKS cells (a gift from Paul Lambert, McArdle Laboratory for Cancer Research, University of Wisconsin) were maintained at subconfluence on mitomycin C-treated J2 3T3 feeder cells in F medium (0.66 mM Ca^2+^) composed of three parts F-12 medium to one part DMEM supplemented with 5% FBS, insulin (5 μg/mL), cholera toxin (8.4 ng/mL), adenine (24 μg/mL), epidermal growth factor (10 ng/mL), and hydrocortisone (0.4 μg/mL), as previously described [33]. J2 3T3 feeder cells were removed from the culture dish by treatment with 0.02% EDTA. To remove the epithelial cells from the culture dish, the monolayer was rinsed with phosphate-buffered saline (PBS) followed by the addition of 0.1% trypsin/0.5 mM EDTA. The cells were then recovered from the suspension by multiple dilutions with E medium and cold PBS, followed by centrifugation.

### 4.2. Generation of NIKS Cell Lines Containing HPV Genomes

Plasmids containing HPV16 and HPV18 genomes were digested with *BamHI* or *EcoRI* to release the whole viral genome. The linearized HPV16 and HPV18 genomes were then re-circularized and purified as described previously [23]. Cells (2 × 10^5^) were seeded in each well of a 6-well plate with incomplete F-media (no EGF). On the following day, the cells were transfected with 1600 ng re-circularized HPV DNA and 400 ng pcDNA6 encoding a blasticidin resistance gene (Invitrogen, Waltham, MA, USA) using FuGENE HD (Promega, Madison, WI, USA). The next day, the cells were seeded onto a 75 cm^2^ flask over blasticidin-resistant feeder cells with incomplete F-media. Cells were selected with 4 μg/mL blasticidin S with complete F-media (with 10 ng/mL EGF) for 4 days and cultured for another 2–3 days in the absence of blasticidin [34]. To overcome the tissue culture issues and to perform our experiments in a more controlled manner, we decided to use cells at passage 2 after transfecting the HPV genome into NIKS cells [24].

The confluency of NIKS cells in culture strongly induces commitment to terminal differentiation [23,24]. Therefore, to induce differentiation, NIKS cells harboring HPV16 or HPV18 were cultured to post-confluent conditions and then collected for further analysis.

### 4.3. Nuclei Preparation

For analysis of the chromatin organization of the HPV16 and HPV18 genomes, cultured NIKS cells harboring HPV16 and HPV18 were washed twice with ice-cold PBS and harvested with a rubber policeman. The pellet was centrifuged at 3000× *g* for 5 min and washed with a buffer containing 10% sucrose, 60 mM KCl, 35 mM HEPES (pH 7.4), 5 mM KH_2_PO_4_, 5 mM MgCl_2_, and 0.5 mM CaCl_2_. The pellet was then suspended in a cold buffer containing 10% sucrose, 60 mM KCl, 35 mM HEPES (pH 7.4), 5 mM KH_2_PO_4_, 5 mM MgCl_2_, 3 mM CaCl_2_, and 15 mM NaCl. After the addition of Nonidet P-40 (final concentration, 0.1%), the samples were pipetted 10 times. To obtain genomic DNA, NIKS cells harboring HPV16 and HPV18 were washed twice with ice-cold PBS and harvested with a rubber policeman. The DNA was then purified using a QIAamp DNA Mini Kit (Qiagen, Hilden, Germany).

### 4.4. MNase Digestion of Chromatin and Purified DNA

The DNA was digested with 1–5 U MNase at 25 °C for 5 min and the reactions were stopped by the addition of 10 mM EDTA, 1% SDS, and proteinase K (800 μg/mL) at 37 °C overnight. The DNA was then purified using phenol and phenol–chloroform extraction and ethanol precipitation.

### 4.5. Southern Blotting

MNase-treated chromatin was examined using Southern blotting. The blot was probed with full-length HPV16 or HPV18 using a Phototope Kit (New England Biolabs, Ipswich, MA, USA). The DNA was loaded onto a 1.5% agarose gel in 0.5× TBE, and the gel was electrophoresed at 50 V for 3 h. The electrophoretically separated DNA was transferred from the agarose gel onto a nylon membrane (Hybond-N+; General Electric, CT, USA) at room temperature overnight. Hybridization was conducted at 42 °C overnight. The bands were detected by chemiluminescent detection and the Phototope-Star Detection Kit (New England Biolabs, Ipswich, MA, USA) (Figure 1).

### 4.6. Nucleosome Scanning Analysis

**Figure 3:**
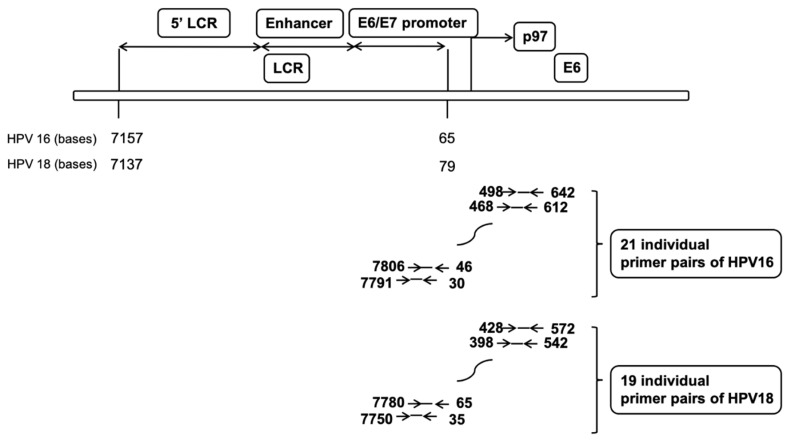
Structure of HPV16/18 including the long control region (LCR) and E6. PCR amplicons were set every 30 bp of the HPV genome spanning the LCR and E6 gene. Each amplicon was 140 bp long including 30-mer primers, and the neighboring amplicons overlapped by 110 bp. The mononucleosomal-sized DNA fragments were quantified by real-time PCR using 21 individual primer pairs of HPV16 or 19 individual primer pairs of HPV18.

MNase-treated chromatin and purified DNA samples were electrophoretically separated on a 1.5% agarose gel; mononucleosome-sized fragments were excised from the gel and purified. The resulting material was analyzed on the ViiA 7 Real-Time PCR System (Life Technologies, Waltham, MA, USA) using Power SYBR Green/ROX master mix (Thermo Fisher Scientific, Waltham, MA, USA) with 15 min denaturation at 95 °C, followed by 45 cycles of 95 °C for 15 s and 60 °C for 60 s. The PCR amplicons were set every 30 bp of the HPV genome, spanning the LCR and the E6 gene. Each amplicon was 140 bp long, including 30-mer primers, and overlapped with its neighboring amplicons by 110 bp (Figure 3).
